# Supplementary material for: Chromothripsis during telomere crisis is independent of NHEJ, and consistent with a replicative origin
Source: Genome Res. 2019 May;29(5):737–49. doi: 10.1101/gr.240705.118 (PMC6499312; doi:10.1101/gr.240705.118)
Supplement: Supplemental Material [file supp_gr.240705.118_Supplemental_file_1.zip › contigs/annotated_contigs/DB84/contig.4.DB84_length_1290_mean_cov_8.48837209302.docx]

**DB84_length_1290_mean_cov_8.48837209302**

```
ATTAAAATTCTGTTTGCTTAGTTTGAACTTTAAAGTGCAATTCCTTTTCTAGTGAAAGATAATAGCTATATTTAATAGTTTAACTTGAA
 >chr5:22530608-22530874 + E=7e-148
CTTTCCAGAAATGACTGAATGAATTTCTTAAATCATCTTAAAACTAACATTAGTGATAAAAATAATAATATATTTTAAAATTCAGAGTT

TTATCATTGGTAACAAGAAGATGTTGTAATTCTATCATCTCAATGCTGTAGTTGTACAAGTAAATAATAAATAACACCACTCAAAC|TT
                                                                                       >
|ATAAAATATTAGCACACCAGAAAAATAATATGTAGATTGCAGGTACAGATGGCAAAAGAAATTCCAATTCACACCAAAGAATCCGGAA
chr5:22531751-22532467 + E=0e+00 p=0e+00
AAGACTCAAAAATGGAGGCAACTGGTACCTTCCAATGTAAGGAAACACACTGGTGTAAAAATAGAAAGATTGGCTGAAAGTCTGTATTA

GAAGCAGCTAGATCCCAAATTCCCTATGATTTCCTGCACAGCCAGAAAGCAACTCCTTCCACACCATGACTGGCAGTTTGAGTTTACAC

CTGGAGACTGCAACGGAGAGTCTCTGGACTGTGGTTCACCAGGCAGGGCTGAAGGCTGGGATGGCATTCTGAGAATGGGGATTATCAGA

GGAATCTCTCAAATCCTGTAAAGTTAGGAAGAAATCGATATCTCAGGGGCTTTGAGGGACAAGTCACATGGAGCTGCATAAGAATAAGA

AATAGGAAATAAGAGAAAATAGGAATAGGAGAGAAAACATTAAGCATGATGTGTACTCAAACACAGCCTGCCTGGCCTTTGGATGCTCG

CCCTGGCACAGTTACACATGGACCTTCTGTTACTGGTCAACTCCTTTGTACGTTTTCAAGGATGTCTGTTAAGAAAGGAATTTTACCTG

CACGACATGGCACATGTGTTAGAGGAGCTATGGTTTGATTTGCTTTGGCATGGGCAGTTCTAACAGATAAATTATATAAATAGGATATA

TAT|TAATTA|ATAAAATATTAGCACACCAGAAA|AATAATA|AAAGCATAAAGGATAAAATATATTAAATAAAAATATAATATTATCA
          >chr5:22531753-22531783 + E=5e-06 p=1e-02 >chr5:22533014-22533295 + E=7e-15
TAGTCCAAAAGTATACAGGGCAAATTACTAGCCTATTTGAAACTTTAAAGAGGTATATCCTGAAAATTTTGTTGCTACTAAAATTATCA
7
AAGAAAATAGATGTTCCCAGGTATTGCACCCTGTCTTTGTTATTTTTTTCTTTTTTCTTTTTCCTTCATCCATTGGCTGTTTTTCTCCT

GTATCAGTGGTTCTCAACAGGGAGAGATTTGACAATGTCAGGGGACATTT
```
